# The Role of Platelet Cell Surface P-Selectin for the Direct Platelet-Tumor Cell Contact During Metastasis Formation in Human Tumors

**DOI:** 10.3389/fonc.2021.642761

**Published:** 2021-03-15

**Authors:** Hans-Åke Fabricius, Sarah Starzonek, Tobias Lange

**Affiliations:** Institute of Anatomy and Experimental Morphology, University Medical Center Hamburg-Eppendorf, Hamburg, Germany

**Keywords:** platelet, metastasis, P-selectin, P-selectin ligand, platelet-tumor crosstalk

## Abstract

Mammalian platelets, devoid of nuclei, are the smallest cells in the blood stream. They are essential for hemostasis, but also transmit cell signals that are necessary for regenerative and generative processes such as inflammation, immunity and tissue repair. In particular, in malignancies they are also associated with cell proliferation, angiogenesis, and epithelial-mesenchymal transition. Platelets promote metastasis and resistance to anti-tumor treatment. However, fundamental principles of the interaction between them and target cells within tumors are complex and still quite obscure. When injected into animals or circulating in the blood of cancer patients, cancer cells ligate platelets in a timely manner closely related to platelet activation either by direct contact or by cell-derived substances or microvesicles. In this context, a large number of different surface molecules and transduction mechanisms have been identified, although the results are sometimes species-specific and not always valid to humans. In this mini-review, we briefly summarize the current knowledge on the role of the direct and indirect platelet-tumor interaction for single steps of the metastatic cascade and specifically focus on the functional role of P-selectin.

## Introduction

Hematogenous metastasis formation of solid human tumors is a multi-step, complex process (1–4) that can be divided into five sequential phases (see below) beginning with distinct changes in individual tumor cells within the bulk primary tumor. Controlled by various tumor cell-intrinsic and external (environmental) factors, future metastatic cells acquire the ability to leave the primary tumor through loosening their cell-cell and cell-matrix contacts, suppressing intrinsic pro-apoptotic stimuli (anoikis suppression), re-arranging their cytoskeleton in the sense of an epithelial-mesenchymal transition (EMT), and acquiring migratory capacity. By releasing matrix-degrading substances, the future metastatic tumor cells, either alone or as cell clusters invade the neighboring stroma and finally enter micro-vessels of the primary tumor (intravasation). Within the bloodstream, the so-called circulating tumor cells (CTCs) can survive for only a few hours due to hostile environmental conditions (shear stress, natural killer (NK) cell attack, apoptotic stimuli due to insufficient anoikis suppression); therefore, they must protect themselves or leave the vascular system as efficiently as possible (extravasation) in order to be eligible for the formation of a later metastasis. For extravasation, tumor cells must overcome the endothelial barrier of the blood vessels, either after dynamic adhesion out of the blood flow (active) or after getting stuck in micro-vessels whose diameters are too small (passive). After leaving the vascular system, these disseminated tumor cells (DTCs) can remain as single cells or small cell clusters for highly variable periods of time (dormancy) before they proliferate again, possibly triggered by the reversal of EMT called mesenchymal-epithelial transition (MET), and thus colonize the foreign stroma to form a clinically manifest metastasis.

The crucial role of tumor cell-mediated platelet activation for promoting different steps of the metastatic cascade, particularly for those taking place within the bloodstream, has extensively been demonstrated and reviewed during the past decade, e.g., (5–13). Nevertheless, anti-coagulant therapy is still not routinely implemented in the treatment of cancer patients (13). Therefore, it is the time to ask which aspects of platelet-tumor interaction have been underestimated so far and whether the common preclinical models for studying the effects of platelets on metastasis formation are really clinically meaningful. This mini-review will briefly summarize the influence of platelets on cancer cell growth in general and on the single steps of the metastasis cascade in particular, taking into account those steps inside and outside the bloodstream, the latter of which have been described less frequently. Moreover, we will specifically deal with the role of P-selectin for the platelet-tumor interaction, highlighting recent advances in the molecular dissection of species-specific differences in the tumor/P-selectin interaction.

## Platelet-Tumor Cell Interaction During the Metastatic Cascade

There is a number of excellent reviews on the complex topic of platelet-tumor-cell interaction available (5, 8, 14–16), which we warmly recommend and at the same time apologize to the authors of a large number of other brilliant studies that we cannot include in this mini-review due to lack of space.

Metastasis is arguably a question of platelets. Most naturally isolated and non-manipulated primary cell lines of both malignant and benign origin can *in vitro* only grow in presence of a growth-promoting supplement, usually serum that contains important active components from platelets. Lysates of platelets and also growth-promoting substances isolated from platelets, such as epidermal growth factor (EGF), fibroblast growth factor (FGF), transforming growth factor β (TGF-ß) or vascular endothelial growth factor A (VEGF-A) can promote and maintain the growth of both tumor cells and primary cells *in vitro* (17–19). This function is mediated by specific receptor tyrosine kinases (RTKs) in the cells. In contrast to serum, platelet-free blood plasma is not able to promote the growth of cells in culture (20). *In vivo*, therefore, growth signaling must either be mediated by growth-promoting substances synthesized in the growing cells themselves or must be replaced by functional changes in the relevant signaling cascades or by the uptake of growth-promoting substances via some kind of mechanism–for instance from platelets. Since proliferation in most cell lines *in vitro* depends on the above-mentioned supplementation by platelet-derived agents, the question arises whether proliferating cells *in vivo* predominantly also require a constant external supply of growth-promoting substances and may therefore largely depend on platelet-derived supplements for proliferation. In any case, it is clear that due to unclear underlying relationships, the validity of experimental results obtained *in vitro* must be assessed with caution before they can be accepted as valid also for the *in vivo* situation.

156 years ago, Armand Trousseau documented that venous thromboembolism (VTE) “phlegmatia alba dolens” is frequently associated with malignant tumors (21, 22). VTE is always associated with platelet activation. Markers of platelet activation are frequently seen in the plasma of cancer patients (23, 24). Tumor microvesicles shed into the blood are able to activate platelets and thereby to initiate blood clotting (25). Half a century ago, Gasic et al. (26) described on the basis of animal experiments that a pharmaceutical reduction of the platelet count leads to a reduction in the number of metastases, drawing attention to platelets as a new option for targeting the problem of metastasis formation. The positive correlation between platelet count and tumor progression is also well-known in clinical oncology (7, 27, 28). Tumors activate platelets and facilitate formation of microclots indirectly via soluble activators, possibly contained in tumor-derived microparticles (MPs) (5, 29), or through direct contact with platelets (30, 31). Platelet activation can lead to thrombosis and VTE (22). Activated platelets adhering to tumor cells are of crucial importance in metastasis formation (5, 15). Blocking this adhesion in experimental models has been proven to be effective in preventing both tumor engrafting and metastasis (14, 32, 33). Integrin aIIbß3 (GPIIb/IIIa) and P-Selectin are two of a number of molecules which have been demonstrated to mediate platelet binding to human tumor cells, both of which can be blocked by heparin (8, 34–38).

**Figure 1:**
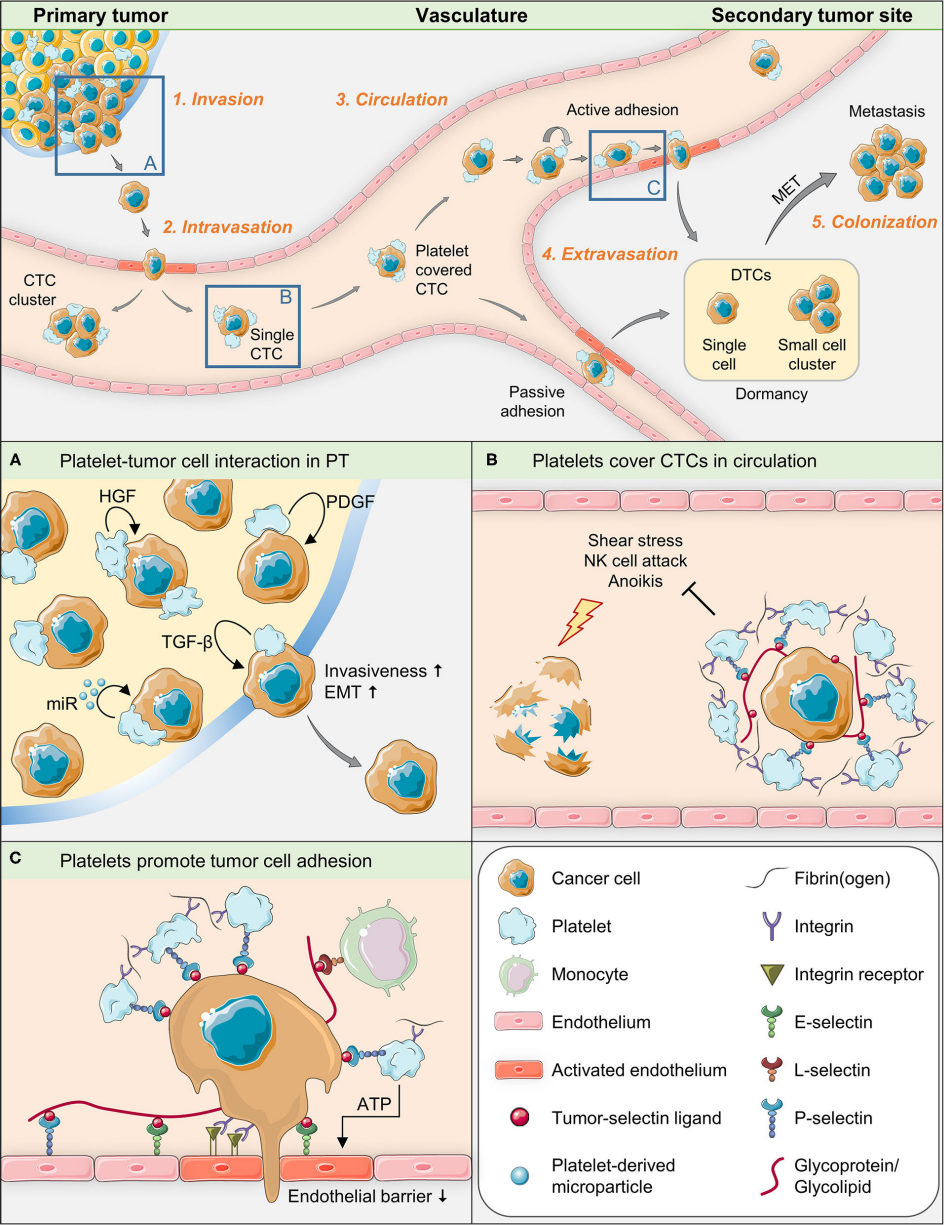
Graphical summary of key aspects of tumor-platelet interaction during single steps of the metastatic cascade.

Platelets are equipped with complete and functional machineries for protein synthesis and for the control thereof (39). Obviously, different signaling events may take place between platelets and target cells, leading to different types of platelet activation. In addition, platelets are able to transfer RNA to recipient cells (40) and thus exert signal-dependent functional influence on them (39). Therefore, it seems likely that the contact between platelets and tumor cells leads to a transfer of platelet contents into tumor cells, including different messenger and microRNAs and growth-promoting factors (41, 42). Platelets have been demonstrated to be able to take up tumor RNA from tumor microvesicles (43) and to profoundly change biological processes in tumor cells, depending on their own sets of genetic information (41). This information changes with platelet provenience, genetic disorders or disease (44). In order to dissect the role of platelets for single steps of metastasis formation, we will divide the entire process into five phases (45) (see Figure 1 for illustration of key aspects of this mini-review):

1. Invasion of the basement membrane and cell migration

In order to invade the surrounding extracellular matrix of the primary tumor, tumor cells have to modify the extracellular matrix, including the basement membrane, and migrate through this matrix to proliferate in the bordering ectopic tissue. A limited number of studies so far directly show that platelets are capable of infiltrating pimary tumors so that platelet-tumor interaction might already take place during this early step of the metastatic process (46–49). When activated, platelets release a variety of growth-promoting agents from their α-granules. Among these are TGF-ß (50, 51), hepatocyte growth factor (HGF) and platelet-derived growth factor (PDGF) that directly promote or help to trigger epithelial-mesenchymal transition (EMT) (52), a crucial process of this first metastatic phase, by which epithelial cells change morphology toward a mesenchymal bipolar type and acquire new metabolic and functional (invasive) capacities (51). In a groundbreaking work, Labelle et al. showed (10) that the absence of TGF-ß in platelets impairs the ability of tumor cells to metastasize due to reduced TGF-ß/ Smad and NF-κB signaling. In order to induce EMT, a tumor cell apparently has to be in direct physical contact with platelets: gene expression signatures associated with EMT and tumor progression were robustly enriched in cells treated with the platelet pellet, but not in cells treated only with the platelet releasate. The importance of direct contact between platelets and tumor cells, also reported in the aforementioned study by Labelle et al., is supported by observations from electron microscopy, where the degranulation of platelets, indicating platelet activation, has proved to be more pronounced in platelets which have immediate contact with tumor cells than in platelets which are located at some distance from them (31, 53). The authors also demonstrated that tumor cells that have direct contact with platelets can engulf parts of these. These facts indicate that tumor cells in the first phase of metastasis depend on at least two signals from platelets (through direct and indirect tumor/platelet contact), whereby signal transmission through platelet-cell contact is mandatory for metastasis formation. Accordingly, a recent study has shown that direct contact between platelets isolated from advanced gastric cancer patients and gastric cancer cells particularly induced migration, invasion, adhesion, and MMP9 expression in the tumor cells (54).

Interestingly, aggregates of extravasated platelets in invasive parts of clinical specimens of human pancreatic cancer biopsies were demonstrated to be associated with markers for the first steps in EMT, such as increased levels of Snail1 and reduced/lost E-cadherin (41, 46). Likewise, platelets directly surrounding primary tumor cells were observed in almost 60% of a Japanese cohort of HER2-negative breast cancer patients (biopsy specimens) and platelet-positive tumor cells showed EMT marker expression (55). Moreover, platelet-derived MPs have been shown to play an active role in cell invasion by transferring microRNA. For instance, microRNA-223 delivered by platelet-derived MPs from NSCLC patients into tumor cells promote invasion by targeting EPB41L3 inside the tumor cells (42). Likewise, platelet MPs derived from ovarian cancer patients promoted EMT and thus migration of epithelial ovarian cancer cells by microRNA-939 (56).

2. Intravasation into the surrounding vasculature or lymphatic system

Growing tumors, like all other cells, depend on blood supply. Pericytes and endothelial cells are important cellular components of the tumor's own vasculature. The proliferation of endothelial cells is dependent on VEGF-A (57). Platelets are the major source of VEGF-A in the blood stream (58, 59). Interestingly, if isolated megakaryocytes are added to endothelial cell cultures, endothelial cell growth can even take place under serum-free culture conditions, i.e., in absence of other extracellular promoters of cell proliferation (60). Platelets are obviously important not only for the proliferation of tumor cells, but also for normal endothelial cells. However, platelets can react selectively to different activation stimuli (61), resulting in differentiated releases of pro- or anti-angiogenic contents from their α-granules. This circumstance explains the ability of platelets to either facilitate or suppress vascularization (62). Therefore, the term “activation” can only be a generic term for a number of finer differentiable processes.

Metastatic dissemination can happen along two paths: Hematogenously, typically by entering the venous route, and along lymphatics. Depending on tumor provenience, vascularization, surrounding tissue and localization, venous intravasation can take place actively or passively (63). Cells having passed EMT are able to pass through the endothelial layer of tumor vasculature into the vascular lumen. Intravasation is also in part facilitated by localized and transient TGF-ß signaling and by the expression of EGF receptors on the tumor cell (63–65). Platelets are important sources of EGF and known to be present in and around extravascular tumor tissue (53) and can thus be presumed to play an active role in this context. Moreover, activated platelets are a primary source of lysophosphatidic acid (LPA) (66), a lipid with growth factor-like signaling properties, which up-regulates the activity of different matrix metalloproteinases in cancer cells (67). These factors promote not only the detachment of tumor cells from the primary site and invasion, but also their enter into the circulatory system (68).

Platelets are absent in the lymphatic vessels, but may be present around tumor tissue in the interstitial space, which is drained through the lymphatic vessels. In case of intravasation into the lymphatic system (69), EMT, which is normally driven by platelets, is not mandatory, the epithelial morphology is usually preserved (70). Instead, a high interstitial (oncotic) fluid pressure in the tumor has been suggested to promote lymphatic intravasation of tumor cells (71).

3. Survival in the circulation

Cancer patients are prone to have a significant number of circulating tumor cells (CTCs) in their blood (72). Such cells are capable to develop into tumors (73). However, most of the shed tumor cells that have entered the bloodstream have been proved to perish, proposedly due to shear stress or attacks by immune effector cells (74). Activated platelets form a protective cloak around CTCs, which is essential for their survival and gives them the ability to metastasize (8). To form the platelet coating, CTCs have receptors and ligands able to bind circulating platelets such as P-selectin ligands (75), whose detailed biochemical composition has long been recognized to be very diverse (76) and is still incompletely known. Activated platelets express a variety of adhesion molecules (8), but P-selectin is one of the most intensely studied in animal experiments (77) and can be inhibited by heparin (32). The platelet cloak obviously provides mechanical protection against shear forces and attacks of the immune system by high-grade transfer of MHC class I molecules to the tumor cell surface (27, 78) and by the TGF-ß-GARP-axis (79). The platelet coating enables CTCs to bind fibrin(ogen) and attract leukocytes, thereby occasionally forming intravascular microclots. Most CTCs circulate as single cells, although CTC clusters have a strongly boosted metastatic potential (80). In addition, the protective coating of platelets provides growth-promoting factors that are essential for the proliferation of tumor cells and tumor vessels. This phase of the metastatic cascade is also of considerable therapeutic interest. Heparins are known to block platelet binding to tumor cells and impair the binding activity of P-selectin (32, 33). They have been shown to impede malignant progression in cancer patients (81) and to prevent the development of transplanted or intravascularly transmitted tumors in experimental mouse models (8, 14, 36, 82, 83). Silva et al. demonstrated in a murine system that an oversulfated non-anticoagulatory heparan sulfate from an ascidian is able to disrupt platelet binding to tumors, thereby preventing metastasis formation (33). This finding suggests that the above properties may be inherent to a whole group of glycosaminoglycans.

4. Extravasation from vasculature to secondary organs

In parallel with the intravasation and circulation of tumor cells, a second, highly complex process takes place: The preparation of the pre-metastatic niche that is mandatory for successful extravasation and metastasis (84–87). A small fraction of the CTCs can in cooperation with cells derived from bone marrow subsequently extravasate into distant organs that have been prepared for extravasation forming a pre-metastatic niche (88). This process is partly driven by tumor-derived MPs and involves a variety of myeloid cells and progenitor cells, platelets, immune cells, the preparatory adaptation of endothelial cells, upregulation of fibronectin, metalloproteinases and several other molecules and reprogramming of stromal cells to form a permissive environment for CTCs (16, 70).

Platelets are known to recruit leukocytes to sites of inflammation in the vasculature. The endothelial adherence of platelets is dependent on Integrin aIIbß3 and on P-selectin (89) and facilitates the formation of metastatic niches. Taken together, platelets have been shown to stimulate extravasation (90, 91), for instance by releasing ATP from their dense granules upon activation, which in turn modulates endothelial junctions and the endothelial cytoskeleton to induce a breakdown of the endothelial barrier (92).

5. Colonization at secondary tumor sites

MicroRNAs regulate several processes in metastasis, such as EMT and stemness of cancer cells (70). In experimental models, EMT has been found to be accompanied by growth arrest (93). As metastatic lesions frequently exhibit epithelial characteristics, and because of known mesenchymal-epithelial transition (MET) in embryonic tissues, a discussion regarding the necessity of MET for colonization, the final step in metastasis, emerged. This discussion produced differentiated results and showed that lymphatic and hematogenic metastasis must be assessed differently with regard to EMT/ MET, as EMT is not mandatory in lymphatic metastasis (70). The role of platelets in colonization of distant organs other than lymphatic must obviously be seen separately. The EMT/ MET landscape was nicely discussed by Jolly et al. (94). In addition, the ability to form blood vessels in distant organs is important for colonization and depends not only on VEGF-A but also on PDGF, which are stored in the α-granules of platelets (95). As the EMT–taking place in primary tumors–obviously “prepares” tumor cells for several subsequent steps of the metastatic cascade, *in vivo* models that circumvent primary tumor formation (such as the most commonly used intravenous or intracardiac injection models) neglect crucial parts of the pathophysiology of metastasis formation (see below).

## The role of P-Selectin for the Platelet-Tumor Cell Interaction

Platelets exert their tumor- and metastasis-promoting effects via membrane components or secreted products, both of which can be stored in their secretory granules. Among the membrane components, P-selectin is one of the most intensively studied mediators of platelet-tumor interaction. Inhibiting this P-selectin-mediated platelet-tumor interaction by heparin attenuates tumor cell dissemination *in vivo* (32). One reason might be that P-selectin critically contributes to the formation of the platelet cloak surrounding CTCs, which protects tumor cells from NK cell attack (27). Furthermore, P-selectin was supposed to mediate the dynamic interaction of tumor cells with platelets by initiating tumor cell tethering and rolling, a process subsequently consolidated into firm adhesion via GPIIb/IIIa (as suggested by *in vitro* experiments on immobilized platelets under shear stress conditions) (96, 97). While P- and L- (leukocyte) selectin have been shown to synergistically affect systemic dissemination of tumor cells *in vivo*, the inhibitory effect of heparin on this process is due to the blockade of P-selectin function (77). P-selectin was also shown to be crucially involved in the release of acid sphingomyelinase from platelets, which was promoted upon tumor cell binding to the platelets through p38 MAPK signaling. Released acid sphingomyelinase in turn was shown to activate integrins on the tumor cell surface promoting metastasis *in vivo* (98). Furthermore, P-selectin also seems to mediate platelet infiltration into tumors through its cytoplasmic domain binding to talin1, thereby triggering talin1-mediated activation of αIIbβ3 integrin and hence recruitment of platelets into tumors (99).

Importantly, however, there are species-specific differences in the regulation of P-selectin levels and in the specificity of P-selectin ligands between mice and men. Pro-inflammatory stimuli such as TNF-α or IL-1β, which are commonly systemically up-regulated in the context of cancer (100), further up-regulate P-selectin mRNA levels in mice (101), but not in humans. Infusing baboons with *E. coli* (leading to markedly elevated plasma TNF-α), increases mRNA levels for E-selectin but decreases mRNA levels for P-selectin in many organs (102). The murine *Selp* gene promoter has canonical binding sites for NF-κB (p50/p52 heterodimers) and ATF-2 similar to those in the *SELE* and *Sele* genes (103). In contrast, the human *SELP* gene promoter lacks these sites (104) and has, instead, a non-canonical binding site for NF-κB (p50 or p52 homodimers) (105, 106). Replacing the murine *Selp* gene promoter with the human promoter leads to higher P-selectin levels on thrombin-activated platelets (107). Likewise, *SELP* transgenic mice also show higher levels of human P-selectin on activated platelets as compared to the normal (mouse) P-selectin levels on activated platelets from wildtype mice (108). These major differences should be carefully considered when the findings of xenograft experiments using human tumor cells within a murine organism are extrapolated to the clinical situation.

Furthermore, not only different P-selectin levels on activated human *vs*. murine platelets, but also different ligands at the tumor cell surface used for binding human *vs*. murine P-selectin have to be considered: as reported quite recently, there is considerably more murine than human P-selectin binding to human tumor cells particularly when tumor cells express both canonical selectin ligands, i.e., the glycan epitopes sialyl-Lewis A and X (sLeA+/X+); there was much less difference seen with sLeA-/ sLeX+ or sLeA-/ sLeX- cells suggesting that the sLeA epitope (capping a variety of different carbohydrate structures) might specifically support murine P-selectin binding (109). All tested tumor cell lines stemming from a range of entities shared the ability to bind human and murine P-selectin, again underlining the crucial importance of P-selectin in the context of cancer. Interestingly, several tumor cell treatments aiming at disrupting tumor cell/ P-selectin interaction impaired human *vs*. murine P-selectin binding quite differently suggesting that different ligands are functional for both species (109). Another dimension of complexity results from the differential binding and adhesion behavior of human tumor cells to murine *vs*. human P-selectin under static and dynamic experimental conditions, respectively: while all tested human tumor cells bound human and murine P-selectin under static conditions, only sLeA+/X+ and sLeX+ cells (but not sLeA-/sLeX- cells) were able to adhere dynamically on murine P-selectin. Among them, only those co-expressing PSGL-1 were able to adhere dynamically on human P-selectin (109). These observations imply that the ability of human tumor cells to directly interact with platelets inside the bloodstream (dynamic conditions) or outside the bloodstream (static conditions) might be remarkably different in mice and men.

Hence, species-specific differences in both the regulation of P-selectin levels on platelets as well as P-selectin binding properties to tumor cells must be considered when extrapolating data from animal models to humans (107, 109). Furthermore, most of our knowledge on the importance of platelets for cancer metastasis stems from experimental metastasis (dissemination) models, in which tumor cells are usually taken from *in vitro* culture for immediate injection into the tail vein or left ventricle of mice (10, 11, 27, 32, 77). This approach abrogates the prior selection of the most metastasis-competent tumor subpopulation, as would be the case within a three-dimensional primary tumor containing heterogeneous stroma components, tumor-infiltrating host cells, regions of differential oxygen and nutrient supply, etc., which all contribute to the selection of a very small fraction of cells that eventually cause metastases. Thus, it is highly questionable to which extent the tumor cells from conventional cell culture (that are used in experimental metastasis models, i.e., intravenous and intracardiac injection models) represent the actual phenotype of tumor cells that would spontaneously detach from a real primary tumor. In addition, intravenous and intracardiac injection (dissemination) models apply huge loads of tumors cells as opposed to the single tumor cells or clusters of few cells that would normally travel through the blood stream during spontaneous metastasis formation, which might imply additional limitations of the very common dissemination models. In the few available studies using spontaneous metastasis xenograft mouse models, P-selectin deficiency alone did not reduce metastasis formation to the lung (110) or intraperitoneally (111). It might therefore well be that some of the previously reported functional *in vivo* experiments on the platelet-tumor interaction during metastasis formation do not really apply to the human situation. This difference might explain why, despite tremendous efforts during the past decades, anti-coagulant therapy is still not routinely used for preventing metastasis in cancer patients. A great step forward would be further mechanistic studies in spontaneous metastasis xenograft models using mice with fully humanized P-selectin expression. It should also be considered that one reason for the suboptimal clinical outcomes may be that anti-coagulants are likely to be ineffective in clinically apparent solid tumors. In these, abnormal metabolic conditions prevail, which, among other things, also lead to necrosis. It is likely that platelet activation and release of growth promoting factors can occur under such conditions without the involvement of specific platelet receptors which can be inhibited by anti-coagulants. Clinical studies using patients with advanced tumor stages are therefore probably not suitable for testing the anti-metastatic potential of anti-coagulants.

An evaluation of nine clinical studies by Akl et al. (112) is similar in content to the consented summary mentioned above (81). An often significant delay of tumor progression, but without clinical cure, is the bottom line. To test the question of prophylaxis against metastasis by heparins, tumor patients with a clinical R0 tumor or patients with a complete remission of non-solid tumors would probably be most appropriate. Non-anticoagulant heparins or heparin analogs could be useful in reducing the risk of bleeding complications in these patients.

In summary, targeting the P-selectin/ligand interaction is a promising approach for the future development of anti-metastatic therapies. To achieve this goal, mice with fully humanized P-selectin should ideally be used in xenograft experiments in the future.

## Author Contributions

H-ÅF and TL both conceptualized, wrote and reviewed the manuscript. SS illustrated key messages and reviewed the manuscript. All authors contributed to the article and approved the submitted version.

## Conflict of Interest

The authors declare that the research was conducted in the absence of any commercial or financial relationships that could be construed as a potential conflict of interest.
